# Long-range epigenetic silencing at 2q14.2 affects most human colorectal cancers and may have application as a non-invasive biomarker of disease

**DOI:** 10.1038/sj.bjc.6605045

**Published:** 2009-04-21

**Authors:** R Mayor, L Casadomé, D Azuara, V Moreno, S J Clark, G Capellà, M A Peinado

**Affiliations:** 1 Institut de Medicina Predictiva i Personalitzada del Càncer (IMPPC), Badalona, Catalonia, Spain; 2 Institut d'Investigació Biomèdica de Bellvitge (IDIBELL), L'Hospitalet, Catalonia, Spain; 3 Institut Català d'Oncologia (ICO), L'Hospitalet, Catalonia, Spain; 4 Cancer Program, Garvan Institute of Medical Research, Sydney, Australia

**Keywords:** DNA methylation, epigenetic silencing, CpG island hypermethylation, diagnostic marker, colorectal cancer, gene silencing, prognostic marker

## Abstract

Large chromosomal regions can be suppressed in cancer cells as denoted by hypermethylation of neighbouring CpG islands and downregulation of most genes within the region. We have analysed the extent and prevalence of long-range epigenetic silencing at 2q14.2 (the first and best characterised example of coordinated epigenetic remodelling) and investigated its possible applicability as a non-invasive diagnostic marker of human colorectal cancer using different approaches and biological samples. Hypermethylation of at least one of the CpG islands analysed (EN1, SCTR, INHBB) occurred in most carcinomas (90%), with EN1 methylated in 73 and 40% of carcinomas and adenomas, respectively. Gene suppression was a common phenomenon in all the tumours analysed and affected both methylated and unmethylated genes. Detection of methylated EN1 using bisulfite treatment and melting curve (MC) analysis from stool DNA in patients and controls resulted in a predictive capacity of, 44% sensitivity in positive patients (27% of overall sensitivity) and 97% specificity. We conclude that epigenetic suppression along 2q14.2 is common to most colorectal cancers and the presence of a methylated EN1 CpG island in stool DNA might be used as biomarker of neoplastic disease.

In mammals, DNA methylation is an epigenetic modification that mainly occurs in the cytosine residue within the CpG dinucleotide, which is underrepresented along the genome. We also find short stretches of CpG-dense DNA, called CpG island, normally free of methylation and frequently associated with the 5′ promoter of many housekeeping or tissue-specific genes (Feinberg and Tycko, 2004; Baylin, 2005; Esteller, 2007a). Promoter CpG islands can become *de novo* methylated in a cancer cell leading to silencing of the associated gene (Baylin, 2005; Esteller, 2007a). Epigenetic inactivation of tumour suppressor genes is a well-characterised mechanism that is found in virtually all types of neoplasms. Many genes that are silenced by promoter hypermethylation in tumours play important roles in carcinogenesis; these include genome stability, cell-cycle entrance, proliferation, apoptosis, etc. (Baylin, 2005; Esteller, 2007a). The analysis of these epigenetic alterations has multiple applications including their use as prognostic factors, early disease markers, and predictors of response to therapy (Laird, 2003; Baylin, 2005; Esteller, 2007a).

Studies to date, using either candidate gene approaches or global surveys have shown that multiple, but discrete CpG islands can be methylated concurrently in any one cancer (Laird, 2003). In a recent study, a genome-wide DNA methylation screening approach showed coordinated hypermethylation of multiple CpG islands spanning a 1 Mb region in colorectal cancer (Frigola *et al*, 2006). It is interesting that, hypermethylation of these CpG islands was accompanied by long-range epigenetic silencing (LRES) of a 4-Mb chromosomal region mapping at 2q14.2 affecting all the genes regardless of whether their promoters were associated with CpG islands and if these were methylated or not (Frigola *et al*, 2006). Beyond this first evidence supporting, the existence of a new mechanism of gene inactivation in cancer, later studies indicate that LRES may also occur in other chromosomal regions and cancers (Stransky *et al*, 2006; Hesson *et al*, 2007; Hitchins *et al*, 2007; Rodriguez *et al*, 2008). This region encompasses the Engrailed-1 (EN1) gene encoding a homeobox transcription factor. EN1 plays a major role in development and upon deregulation in neoplasia (Bachar-Dahan *et al*, 2006; Rauch *et al*, 2007).

This work aims to determine the extent and prevalence of gene silencing in the chromosomal region 2q14.2 in human colorectal cancer and its possible association with the clinicopathological features of the tumours. We have also investigated the utility of one of the hypermethylated genes (EN1) as a diagnostic marker in stool and serum DNA samples of colorectal cancer patients.

## Materials and methods

### Samples

A series of 108 patients pre-operatively diagnosed as having colorectal cancer and operated upon with curative or palliative intention between 1996 and 1998 at the Ciutat Sanitària i Universitària de Bellvitge was used. This series was part of a larger collection of patients prospectively included in a study designed to evaluate the prognostic value of genetic and epigenetic alterations (Gonzalez-Garcia *et al*, 2000; Vendrell *et al*, 2005). DNA methylation profiles were analysed in all 108 tumours and their normal tissue counterpart. A subgroup of 17 carcinomas were microdissected and the tumour infiltration front with >75% tumour cell content, as assessed by visual examination of hematoxylin–eosin stained preparations, was selected for gene expression analysis. Special care was taken to discard areas with necrotic tissue or harbouring a high-inflammatory component. Clinicopathological information was available for 91 cases (see Supplementary Table 1). Mean follow-up was 79.9±20.3 months. Additionally, 10 colorectal adenomas were obtained from the Hospital de la Santa Creu i Sant Pau (Barcelona, Spain). In all cases, surgical specimens were collected in the operating room and immediately taken to the Pathology Department in ice. Carcinomas and paired normal samples were snap frozen within 2 h after removal and then stored at −80°C.

To study EN1 methylation as a diagnostic marker in colorectal cancer a different series of 30 patients with available DNA from normal and tumour tissues, stool and serum was used. Additionally, stool and serum DNA was available from 30 healthy donors matched by age and sex with the patient group. Stool DNA was extracted from cellular material obtained after centrifugation of bowel lavage or solid stools as described earlier (Puig *et al*, 1999). Serum DNA was obtained as described earlier (Castells *et al*, 1999).

The study protocol was approved by the Ethics Committee. No chemo- or radiotherapy was given prior to surgery. DNA and RNA amenable for genetic analyses were obtained using standard procedures.

### DNA methylation analysis

#### Bisulfite sequencing

Bisulfite conversion was carried out using 2 *μ*g of DNA for 16 h at 55°C under conditions described earlier (Stirzaker *et al*, 2004). A nested PCR was carried out in all cases to obtain a fragment of each one of the CpG islands to be analysed (see primer sequences in Supplementary Table 2). Bisulfite sequencing was carried out using the BigDye Terminator cycle sequencing kit (Applied Biosystems, Carlsbad, CA, USA). The degree of methylation was calculated by comparing the peak height of the cytosine signal with the peak height of the cytosine plus thymine signal as described (Melki *et al*, 1999).

#### Real-time PCR temperature dissociation (melting curve (MC))

Bisulfite conversion and PCRs were carried out as for bisulfite sequencing, except for the nested PCR, that was carried out using a Light Cycler 2.0 (Roche, Mannheim, Germany) and the Fast Start DNA Master Sybr Green I mix (Roche) with real-time detection as described (Frigola *et al*, 2006). Melting curves (MC) from fully unmethylated samples (as determined by bisulfite sequencing) were used as controls.

#### Methylation specific PCR (MSP)

Bisulfite conversion and PCRs were carried out as for bisulfite sequencing, except for the nested PCR, which was carried out using specific primers for methylated and unmethylated DNA (Supplementary Table 2).

### Gene expression analysis

cDNA was obtained by retrotranscription of 500 ng of RNA with M-MLV retrotranscriptase (Invitrogen, Carlsbad, CA, USA) using random hexamers (Amersham Biosciences, Chalfont St Giles, UK) at 37°C for 1 h. cDNA levels were quantified using the Light Cycler 2.0 real-time PCR system with Fast Start Master SYBR Green I kit (Roche). For a 10 *μ*l PCR reaction volume, 1 *μ*l of cDNA and 9 *μ*l of mastermix were added to each capillary. Mastermix was prepared to a final concentration of 3.5 mM MgCl_2_ and 0.5 *μ*M of each primer. Primers used for expression analysis are listed in Supplementary Table 3.

### Statistical analysis

All results are expressed as a mean±s.d. Statistical differences between variables were analysed with unpaired/paired *t*-tests or analysis of variance (ANOVA), as appropriate. Contingency tables were analysed by the χ^2^ or Fisher's-exact test. Survival curves were traced according to the Kaplan–Meier method. The statistical significance between survival curves was tested using the log-rank test. All *P*-values are calculated from two-sided statistical tests.

## Results

### Frequency and extent of 2q14.2 CpG island methylation in human colorectal carcinomas and adenomas

**Figure 1:**
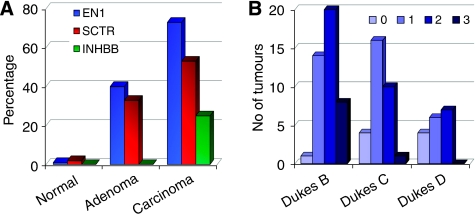
DNA methylation across 2q14.2 in colorectal cancer. (**A**) Frequencies of the methylation in EN1, SCTR and INHBB CpG islands in normal colonic mucosa, and colorectal adenomas and carcinomas. Y-axis indicates the percentage of samples with methylation for each gene. (**B**) Incidence of methylation in colorectal carcinomas according Dukes' stage. Y-axis indicates the number of tumours exhibiting methylation in 0, 1, 2 or 3 of the CpG islands analysed (EN1, SCTR and INHBB).

The three CpG islands associated with the promoter region of the genes EN1, SCTR and INHBB mapping to 2q14.2 have been identified earlier to be hypermethylated in colorectal cancer (Frigola *et al*, 2006). These islands have been characterised by direct bisulfite sequencing and MC analysis in an extended series of 108 colorectal carcinomas with their paired normal tissue and in 10 adenomas. Two more CpG islands corresponding to genes PTPN4 and RALB were also analysed but remained unmethylated in all the normal and tumour samples (data not shown). An excellent agreement between results obtained by bisulfite sequencing and MC analysis was observed. Illustrative examples of non-methylation, and partial and full methylation are shown in Supplementary Figures 1 and 2. Methylation of any of the CpG islands analysed was very rare in normal colon mucosa (only three cases; Supplementary Table 4) and always appeared as a low percentage of methylated molecules. Clonal analysis of normal and tumour samples with partial methylation showed the coexistence of densely methylated with poorly methylated molecules in all cases, confirming the presence of cell populations with heterogeneous DNA methylation profiles (data not shown). On the other hand, most carcinomas (82 out of 91, 90%) exhibited methylation of at least one gene (Supplementary Figure 3A), with EN1 as the most frequently methylated gene in both carcinomas (66 out of 90, 73%) and adenomas (4 out of 10, 40%); SCTR also presented a high level of methylation in carcinomas (48 out of 90, 53%) and adenomas (3 out of 10, 33%); whereas INHBB showed the lowest rate of methylation (23 out of 91 carcinomas, 25%, and none of the 10 adenomas) (Figure 1 and Supplementary Table 4).

The methylation status of three additional CpG islands (CpG104, CpG 41 and CpG 173) flanking the EN1 CpG island (CpG128) (see Supplementary Figure 3B) was determined in a subset of 23 colon carcinomas and their matched normal mucosa from the series of 108 cases by PCR MC analysis. For each gene, a subset of 50% of the samples were re-analysed by bisulfite sequencing and all results were confirmed. As expected, none of the normal mucosa samples exhibited methylation in any of the six CpG islands analysed. In this subgroup, SCTR CpG island (CpG67) was the most frequently methylated (21 out of 23, 91%), followed by CpG173 (20 out of 23, 87%) and EN1 (17 out of 23, 74%) (Supplementary Figure 3C). The methylation status was independent for all the CpG islands analysed (data not shown) and no correlation between the methylation frequency and CpG island size, CpG content, G+C content, or CpG observed/expected ratio was observed.

### Genes at 2q14.2 are down-regulated in most colorectal tumours

**Figure 2:**
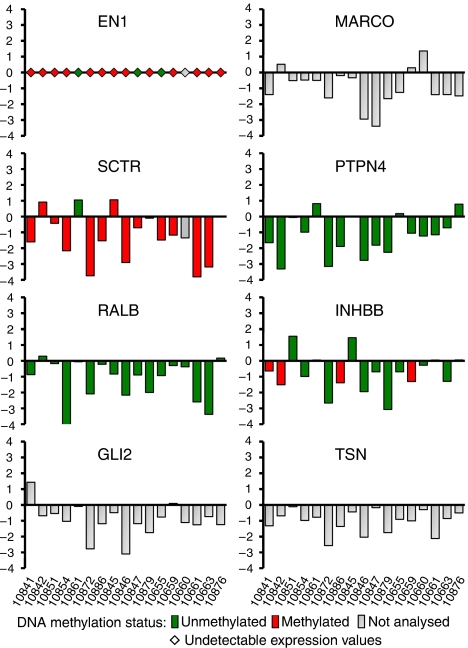
Relative expression levels of the eight genes analysed across the 2q14.2 chromosome region in 17 colorectal tumour tissues and their paired normal mucosa. The log2 of the tumour/normal ratio is represented. Negative values indicate downregulation in the tumour as compared with the respective normal tissue. Expression levels were normalised using the 18S rRNA expression as control. Methylation status was determined using melting curve (MC) analysis and is depicted in column filling as indicated. Grey column indicates genes without promoter CpG island (MARCO, GLI2) or not analysed (TSN).

To get insights into the extent of the gene suppression and its association with promoter hypermethylation, we analysed by real-time PCR the expression of eight genes mapping to 2q14.2 region in an independent series of 17 colorectal tumour samples and their matched normal pairs. Tumour tissues were microdissected to minimise the presence of non-tumour cells in the sample. The methylation state of five of the eight genes was determined using the MC analysis. EN1 expression was undetectable in all the samples. In agreement with earlier observations in pooled samples (Frigola *et al*, 2006), most of the genes were down-regulated in all the tumours as compared with their paired normal mucosa (Figure 2). Interestingly, suppression affected the genes with methylatable CpG island (SCTR and INHBB) irrespective of its methylated state, genes with unmethylated CpG island (PTPN4, RALBB, TSN), and genes without CpG island (GLI2, MARCO). These results indicate that epigenetic suppression of the region is a very frequent event in colorectal cancer and suggest that DNA hypermethylation of some of the CpG islands is a secondary manifestation of the silencing.

### Genetic and clinicopathological features of the colorectal tumours with and without methylation in EN1, SCTR and INHBB

**Figure 3:**
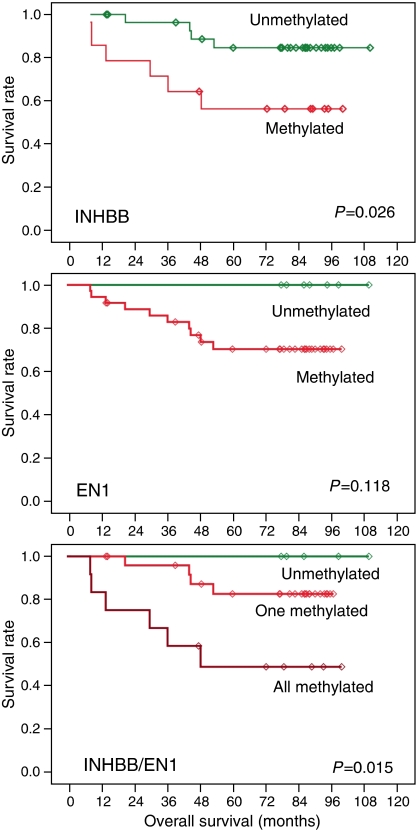
Overall survival in colorectal cancer patients according to the methylation status of INHBB and EN1. Tumours with methylated INHBB or EN1 showed a reduced survival rate, but differences reached only statistical significance for INHBB or the combination of both, INHBB and EN1.

Next, we wondered whether the methylation status of each one of the CpG islands analysed might be associated with genetic and clinicopathological characteristics of colorectal cancer patients. Sex, Dukes' stage, age, tumour localisation, the presence of mutations in the p53 and K-ras genes and microsatellite instability (MSI), and follow-up parameters were considered together with the methylation status of the EN1, SCTR and INHBB CpG islands in the series of 91 patients. Early stage tumours showed a higher proportion of methylated genes (*P*=0.020) (Figure 1B and Supplementary Table 1) and SCTR was the gene exhibiting the higher differences (*P*=0.015). In a subset of 50 of the 91 cases, chromosome profiles determined by comparative genomic hybridisation were available (Vendrell *et al*, 2007). No associations with specific chromosomal aberrations or with global indicators of chromosomal instability were observed. Regarding disease outcome, the number of methylated genes did not seem to modify the survival rate (data not shown). At the single gene level, patients with methylation at INHBB or EN1 showed a worse overall survival, although the differences were only statistically significant for INHBB and when the methylation status of both promoters was combined, in which case differences were more prominent (Figure 3).

### Detection of EN1 methylation as a diagnostic tool in colorectal cancer

**Figure 4:**
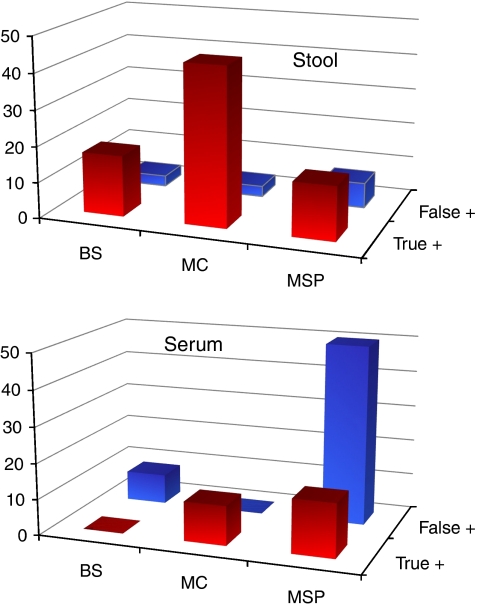
Detection of EN1 methylation in stool and serum DNA as a diagnostic marker of colorectal cancer. ‘True positive’ indicates the percentage of all colorectal cancer patients with a methylated marker in both the tumour and the test sample (stool or serum DNA). ‘False positive’ corresponds to the percentage of healthy individuals with a methylated result in the test sample. The best score was obtained when melting curve (MC) analysis was applied to detect EN1 methylation in stool DNA (27% of overall sensitivity, 44% sensitivity in positive tumours, and 97% specificity).

The elevated methylation frequencies of EN1 in colorectal adenomas and carcinomas lead us to evaluate its possible application as a diagnostic marker of colorectal cancer. We determined the methylation status of EN1 CpG island in the DNA obtained from stools, serum and the corresponding tumour samples in an independent series of 30 colorectal cancer patients. Stools and serum DNA obtained from 30 healthy individuals paired by age and sex were used as control group. We applied different techniques to determine the methylation status, namely direct bisulfite sequencing (BS), analysis of MCs and methylation-specific PCR (MSP) (Supplementary Figure 4). To determine the true positive and false positive obtained with each technique, the result obtained from the stool or serum DNA was compared with that from the respective tumour using the same technique. Full data set is provided in Supplementary Figures 5–9 and Supplementary Tables 5 and 6. Samples exhibiting ⩾25% methylation (depicted as light grey in figures) in at least half of the analysed CpG sites or ⩾50% methylation (depicted as dark grey dots in figures) in at least 1/4 of the analysed CpG sites were considered as methylated. MC analysis appeared as the most efficient method to detect EN1 methylation in stool DNA with a sensitivity of 44% (8 out of 18) and 27% (8 out of 30) considering patients with methylated tumours or all patients, respectively (Figure 4 and Supplementary Table 5). In patients with methylation in the tumours, 17% (3 out of 17) were detected by bisulfite sequencing and 14% (4 out of 27) by MSP. In serum, the sensitivity of MC analysis was lower (2 out of 18, 11%). All samples from healthy donors were unmethylated as determined by MC analysis but with a single exception in one stool DNA. Bisulfite sequencing of this sample showed full methylation in three CpGs and full unmethylation in the rest of CpG sites. As two of these methylated CpG sites were located in the priming region used for MSP analysis, neither methylated nor unmethylated product was obtained using this technique (Figure 4 and Supplementary Table 6). Altogether, MC analysis appears to be the most accurate method to detect the presence of EN1 methylation in stool DNA.

## Discussion

Here we report the high prevalence of coordinated epigenetic silencing at 2q14.2 in most if not all colorectal carcinomas. We also show that hypermethylation is an early event that can be selected for during tumourigenesis. This is in concordance with studies at genomic level showing the accumulation of hypermethylation during tumour progression (Toyota *et al*, 1999; Frigola *et al*, 2005). The commonness of LRES in colorectal cancer has been confirmed in independent studies, that have also noted the association between hypermethylation of the genes in the 2q14.2 region and other CpG islands (Karpinski *et al*, 2008).

The gene expression down-regulation in tumour affected all genes, irrespectively of the presence of CpG island or the methylation status, confirming the earlier results obtained in cell lines and pooled normal and tumour DNAs (Frigola *et al*, 2006). The suppression of 2q14.2 in all the tumours analysed stresses its high prevalence in colorectal cancer. Moreover, the lack of association with DNA methylation suggests that the latter is a secondary phenomenon (Mutskov and Felsenfeld, 2004; Stirzaker *et al*, 2004; Strunnikova *et al*, 2005). The arbitrary distribution of DNA methylation along the methylatable CpG islands (Supplementary Figure 3) is also consistent with a model in which regional suppression is followed by spurious DNA methylation of silenced CpG islands showing appropriate epigenetic signatures (Stirzaker *et al*, 2004). It is of note that some of the genes exhibited wide variations in their expression levels in normal colonic mucosa. This implies that contaminating non-tumour cells may mask the downregulation in tumour samples and, hence, microdissection is critical to obtain a precise assessment of differential expression.

The methylation state of the gene promoters did not seem to be associated with clinicopathological or genetic features of the tumours. Some trends were observed, but of little significance and might result from the performance of multiple statistical tests. The large prevalence of gene suppression across 2q14.2 and the limited accuracy of CpG island hypermethylation as a surrogate marker of gene silencing seem to hinder this study. Therefore, the implications of this alteration in colorectal cancer remain unknown and future studies at cellular level are required to determine its causes and consequences. On the other hand, the possible association of EN1 and INHBB methylation with worse outcome in early stages of the disease deserves further investigation, as it might have a prognostic value in a subgroup of patients that may benefit from more aggressive therapies.

DNA testing in non-invasive samples seems to be a feasible approach that can complement and probably outperform other screening tests for colorectal cancer (Dong *et al*, 2001; Ahlquist, 2002; Traverso *et al*, 2002), although its routine clinical application is still under debate (Brenner and Rennert, 2005; Capella, 2005). *De novo* DNA methylation in multiple CpG islands is a common event in cancer and a large number of new tumour biomarkers have appeared as promising candidates (Esteller, 2007b). Detection of CpG island methylation in human DNA isolated from stool (Belshaw *et al*, 2004; Leung *et al*, 2004; Chen *et al*, 2005; Zitt *et al*, 2007) or serum (Zou *et al*, 2002; Leung *et al*, 2005; Nakayama *et al*, 2007; Lofton-Day *et al*, 2008) has been proposed as a new strategy for the early diagnosis of colorectal neoplasia. Other studies with comparable series have reported high sensitivities for different methylation markers used alone (Chen *et al*, 2005; Lenhard *et al*, 2005; Huang *et al*, 2007a; Wang and Tang, 2008) or in combination (Leung *et al*, 2004; Petko *et al*, 2005; Huang *et al*, 2007b; Lofton-Day *et al*, 2008), although a wider application is usually hinder by a limited specificity.

The high prevalence of methylation in EN1 CpG island (three out of every four carcinomas show hypermethylation of this gene) together with its possible functional role in cancer (Bachar-Dahan *et al*, 2006; Rauch *et al*, 2007) lead us to evaluate its putative clinical usefulness as a diagnostic marker of disease. Of the three techniques used, MSP seems to be extremely sensitive but results in a high rate of positive results in healthy subjects. MC analysis appeared as the best alternative based on its simplicity and performance: 97% specificity and 44% sensitivity in patients with a methylated EN1 in the tumour. This corresponds to a 27% overall sensitivity when non-informative patients (showing unmethylated EN1 in the tumour) are considered. As shown in dilution experiments (Supplementary Figure 2), dissociation temperatures for partially methylated DNA can be distinguished from unmethylated DNA even when just a fraction (10%) of the cells show methylation. Although the short number of cases analysed precludes a definitive conclusion, the diagnostic utility of EN1 methylation by itself or as part of a panel of biomarkers deserves further consideration and evaluation in large series of cases.

In summary, our study shows the high prevalence of epigenetic suppression along 2q14.2 in colorectal cancer. This suppression is manifested in biopsied tumour samples as global downregulation of all the genes mapping to this region and DNA methylation of several CpG islands as a likely secondary event. These observations confirm that long-range epigenetic silencing across 2q14.2 is a feature of most colon cancers. Finally, the high prevalence of EN1 CpG island methylation makes this alteration a good candidate as a non-invasive early diagnostic target. Our preliminary analysis indicates the feasibility of the detection of methylated EN1 promoter in stool DNA as a marker of disease.
