# Supplementary material for: Long-range epigenetic silencing at 2q14.2 affects most human colorectal cancers and may have application as a non-invasive biomarker of disease
Source: Br J Cancer. 2009 Apr 21;100(10):1534–9. doi: 10.1038/sj.bjc.6605045 (PMC2696749; doi:10.1038/sj.bjc.6605045)
Supplement: Supplementary Figures 1–10 [file 6605045x1.pdf]

Long range epigenetic silencing in 2q14.2 affects most human colorectal cancers and may have application as a non-invasive biomarker of disease

Regina Mayor, Laura Casadomé, Daniel Azuara, Victor Moreno, Susan J. Clark, Gabriel Capellà, Miguel A. Peinado

Supplemental Information

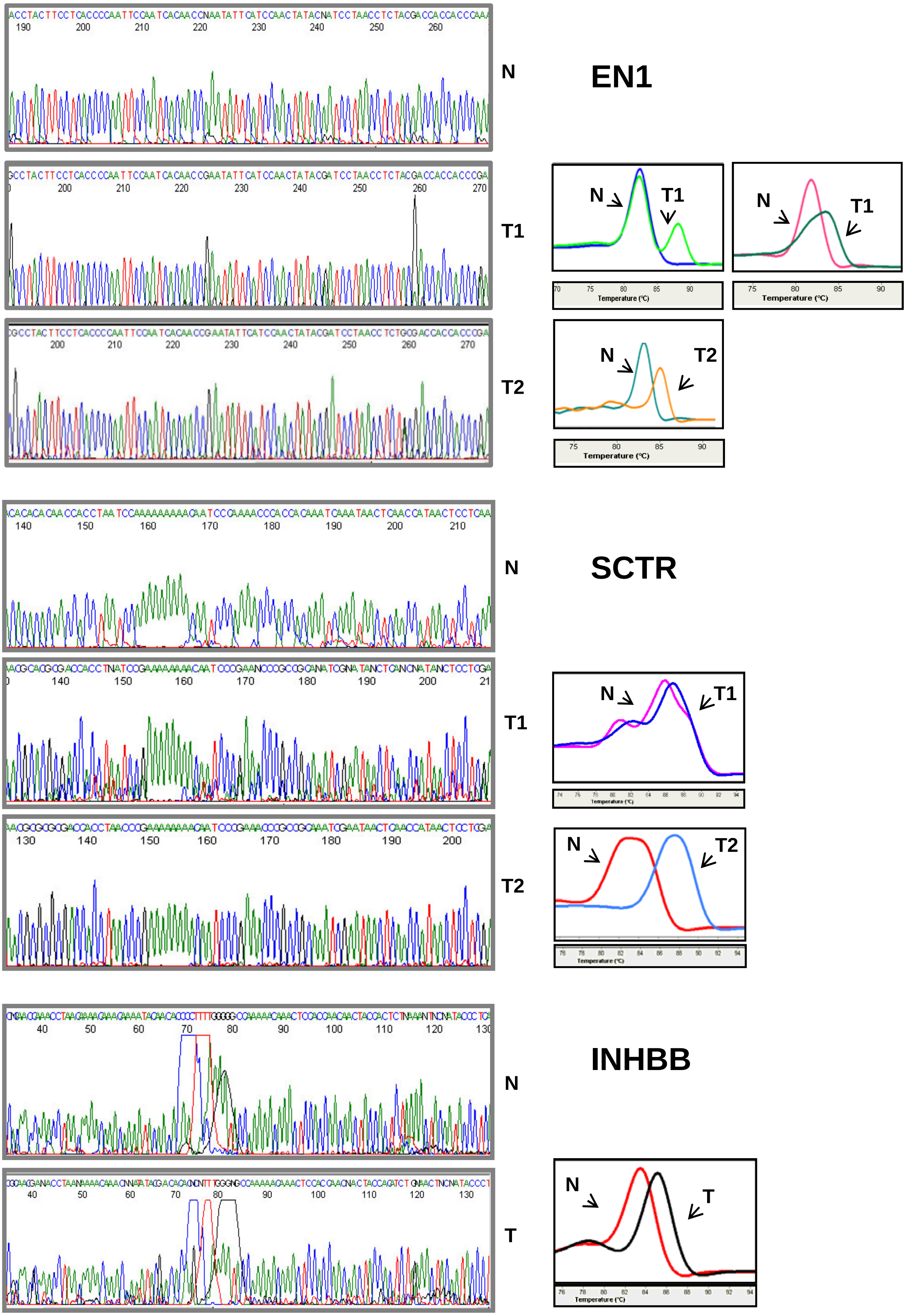

Supplemental figure 1. Methylation profile of EN1, SCTR and INHBB genes by direct bisulfite sequencing and Real-Time PCR dissociation analysis in paired normal (N) and colon carcinomas (T). Illustrative examples for fully methylated, partially methylated and unmethylated sample DNAs.

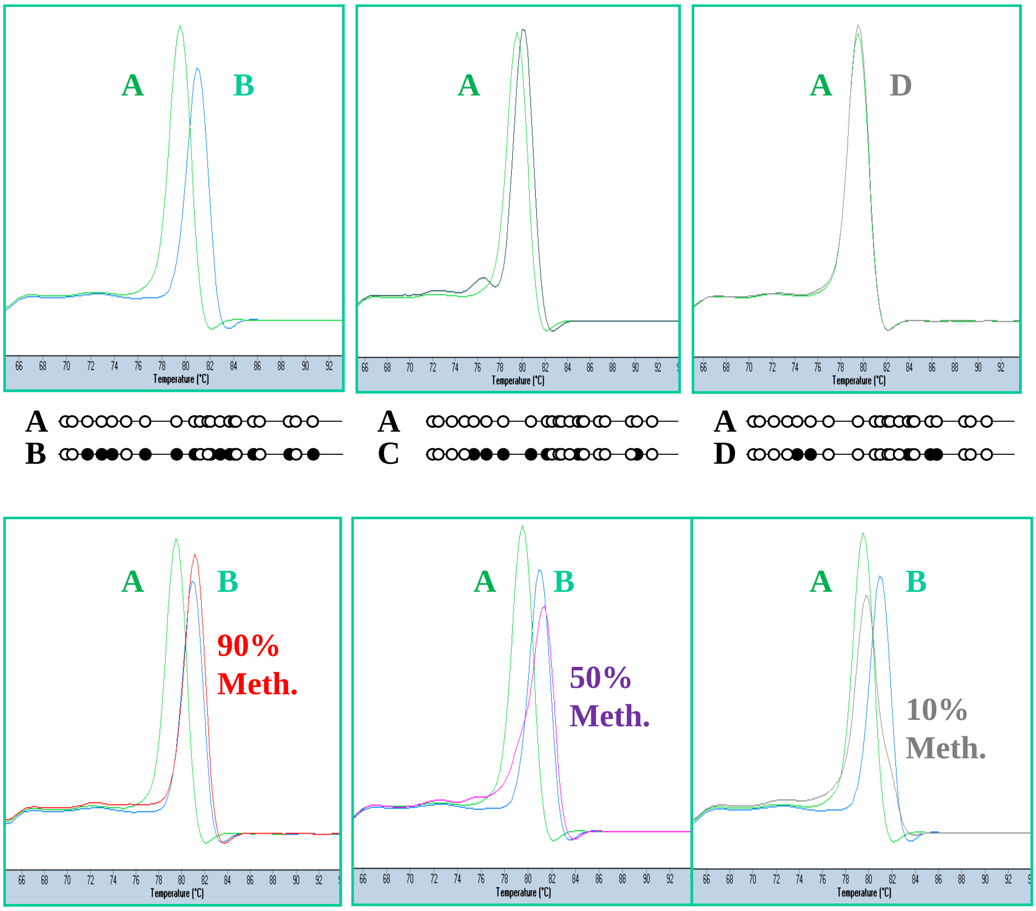

Supplemental figure 2. Melting curve methylation analysis in pure clones and mixed clones of EN1 bisulphite PCR product. Methylation profiles of four different clones (A, B, C, D) of EN1 are represented by melting curve and bisulphite sequencing analyses in the upper part. Empty dots represent unmethylated CpG sites; black dots represent methylated CpG sites as determined by DNA sequencing. Clone A: 0 methylated CpG sites; clone B: 12 methylated CpG sites; clone C: 7 methylated CpG sites; clone D: 5 methylated CpG sites. Methylation profiles of three different mixes (9:1, 1:1, 1:9) of the completely unmethylated clone A and the methylated clone B are represented together with the pure clones A and B. Dilution curves are shown as the percentage of methylated into unmethylated DNA.

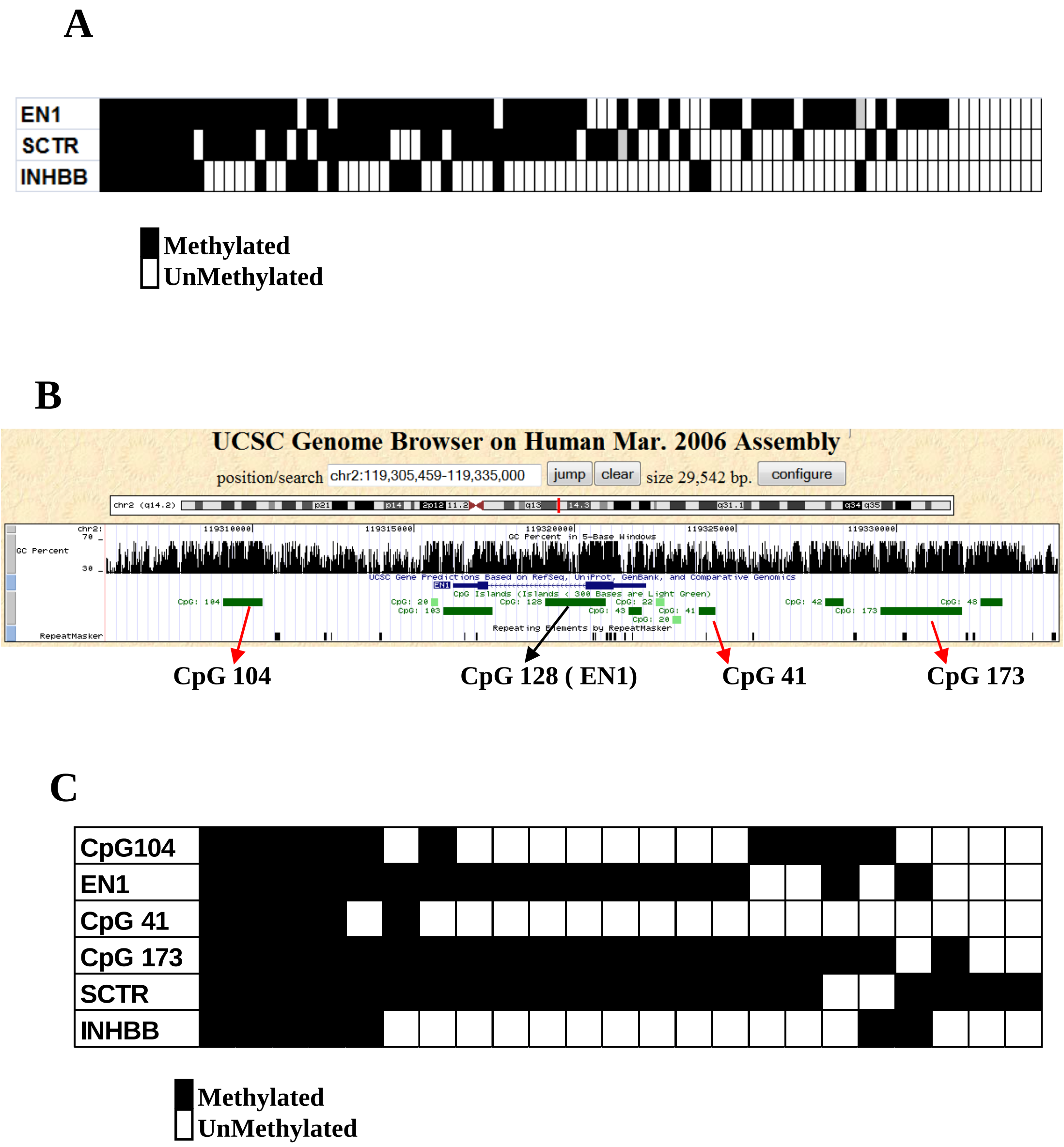

Supplemental Figure 3. Extent of methylation across 2q14.2 region. **(A)** DNA methylation in EN1, SCTR and INHBB CpG island promoters in a series of 91 colon carcinomas. (B) UCSC genome browser window illustrating the position of three additional CpG islands in regard to the EN1 gene. (C) DNA methylation in six CpG islands across 2q14.2 in a subgroup of 23 colorectal carcinomas.

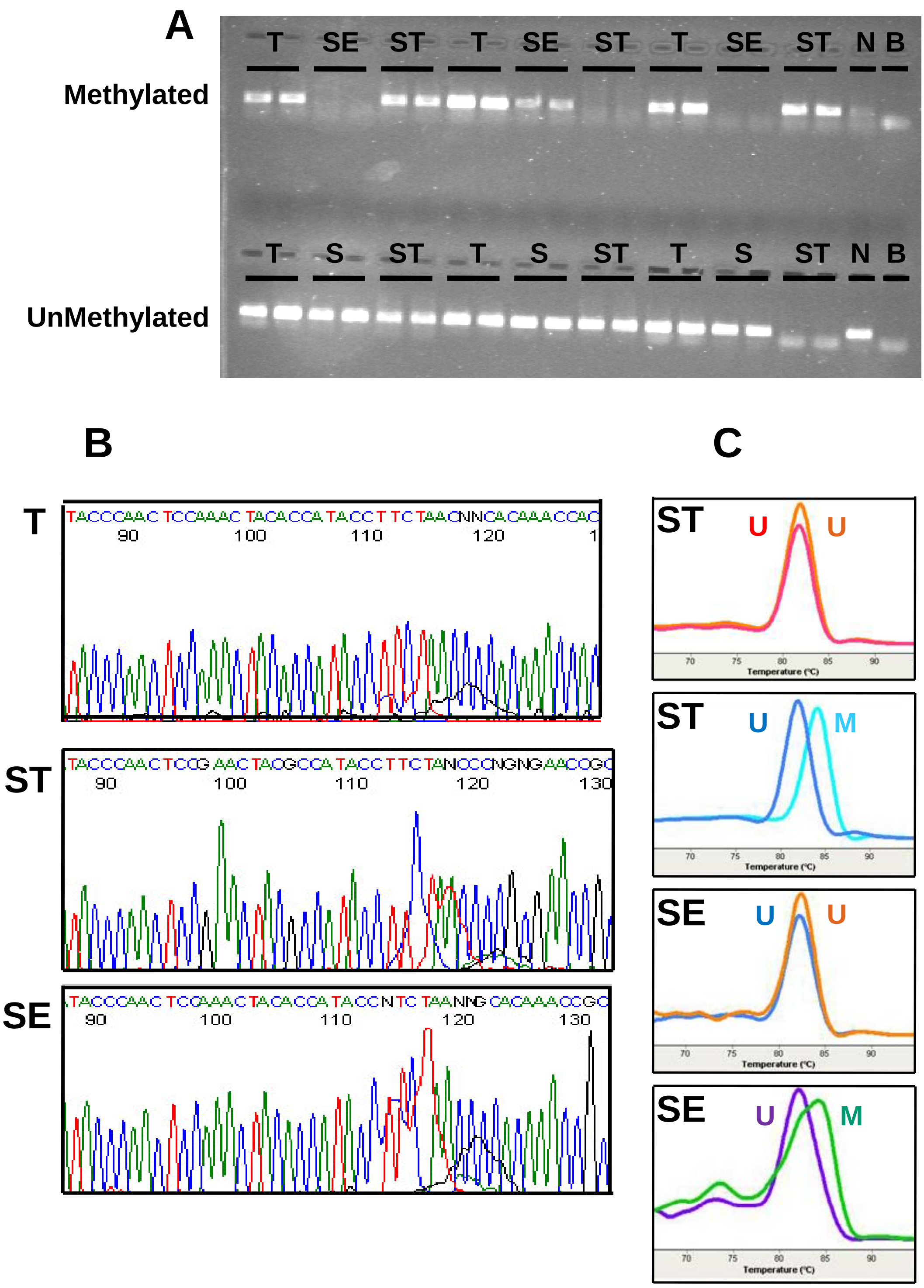

Supplemental Figure 4. Illustrative examples of DNA methylation analysis of the EN1 CpG island in tumor, stool (ST) and serum (SE) DNA using MSP (A), direct bisulfite sequencing (B) and melting curve analysis (C). Normal tissue DNA (**N**) and a blank (B) control were also included.

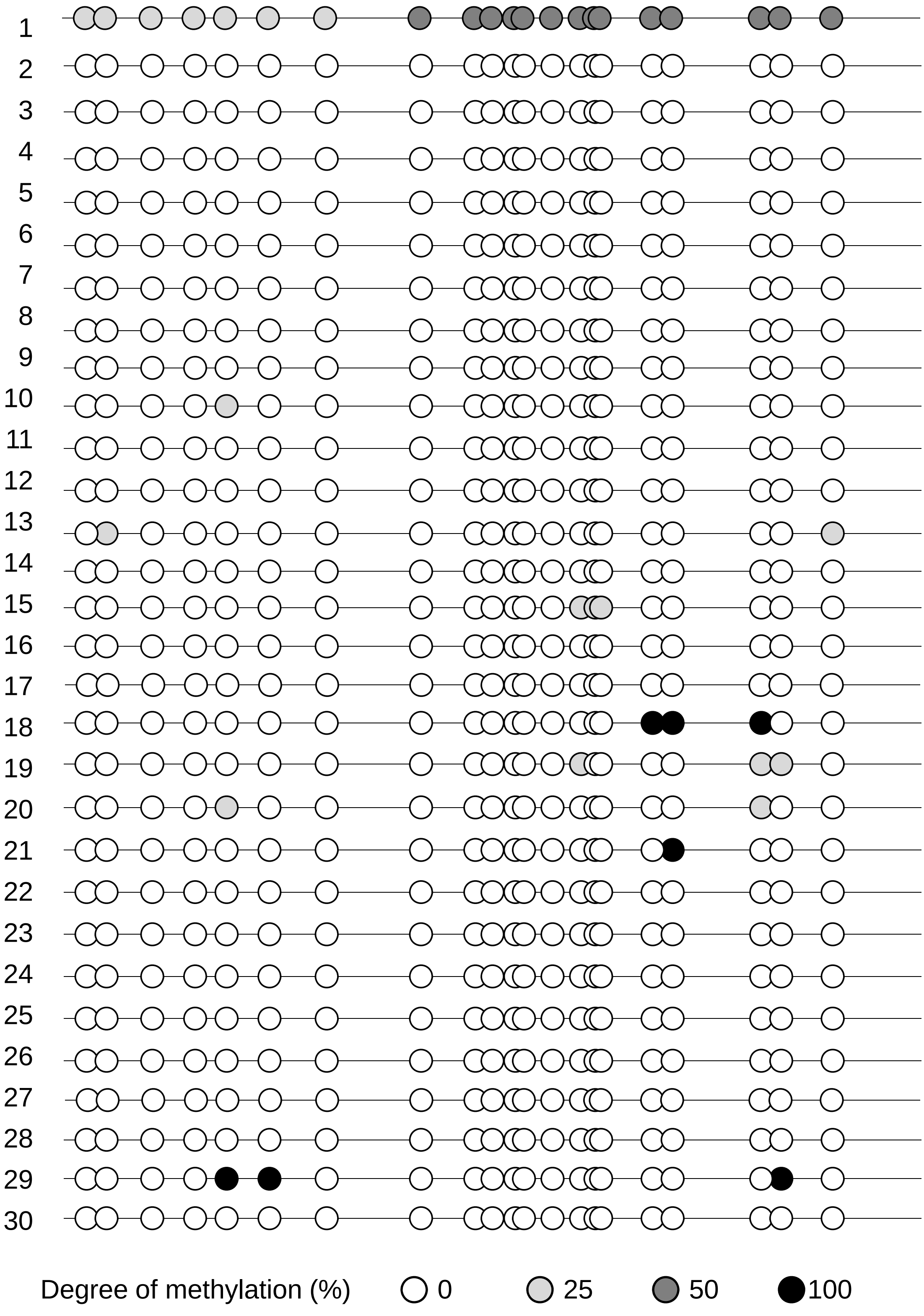

Supplemental Figure 5. Direct bisulfite sequencing data for EN1 CpG island promoter gene in the stools of 30 healthy individuals.

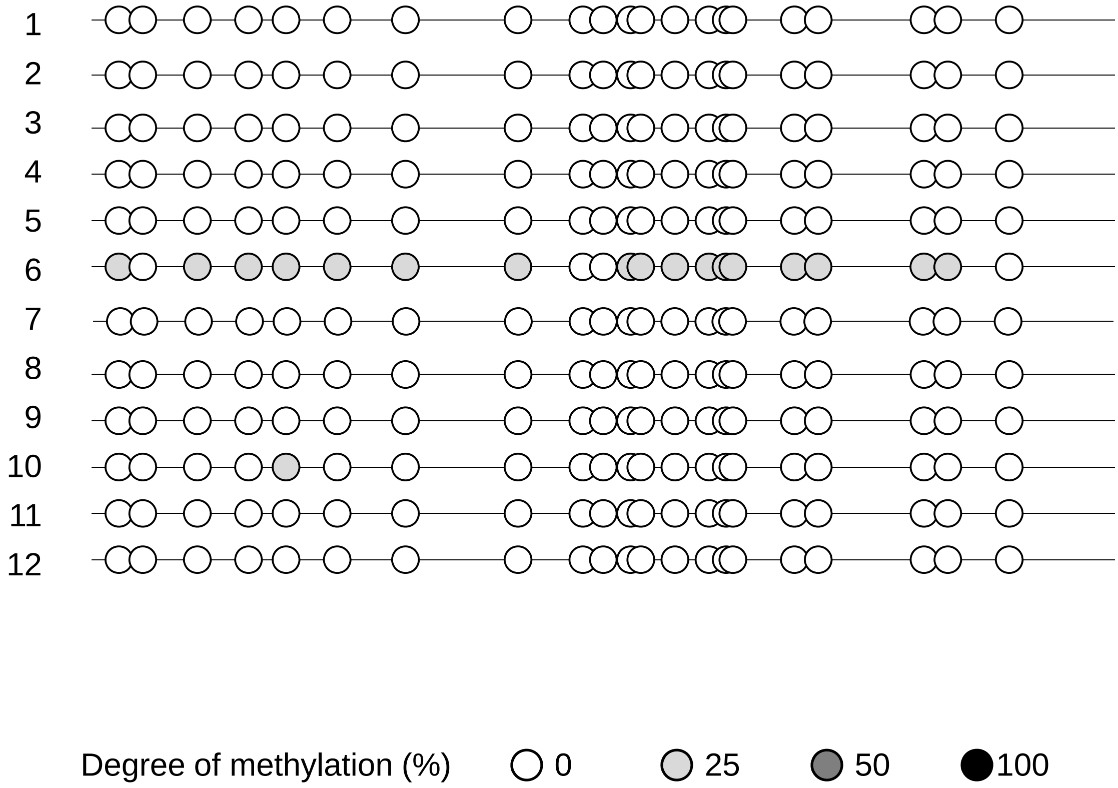

Supplemental Figure 6. Direct bisulfite sequencing data for EN1 CpG island promoter gene in the serum of 12 healthy individuals.

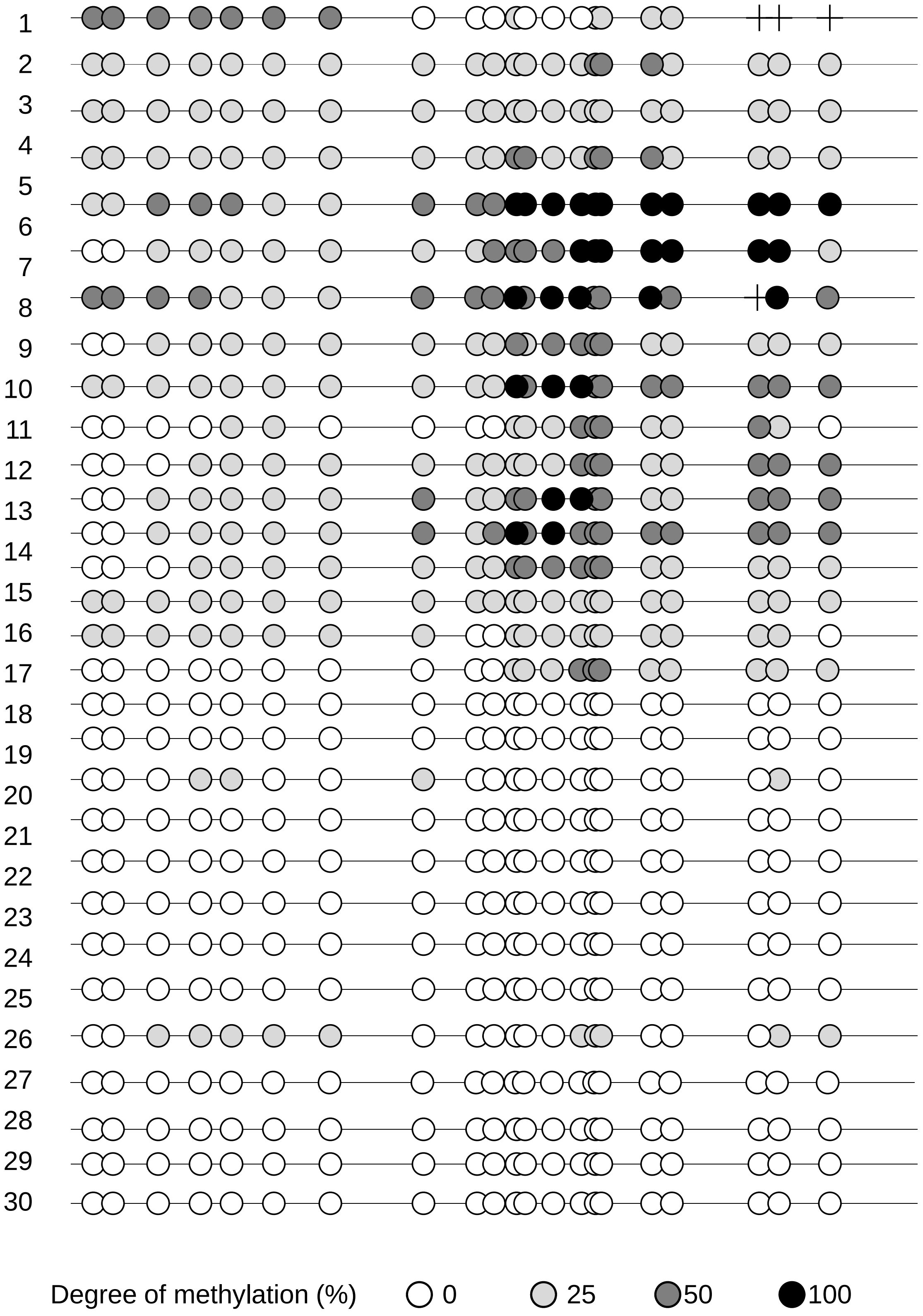

Supplemental Figure 7. Direct bisulfite sequencing data for EN1 CpG island promoter gene in the tumor of 30 colorectal cancer patients.

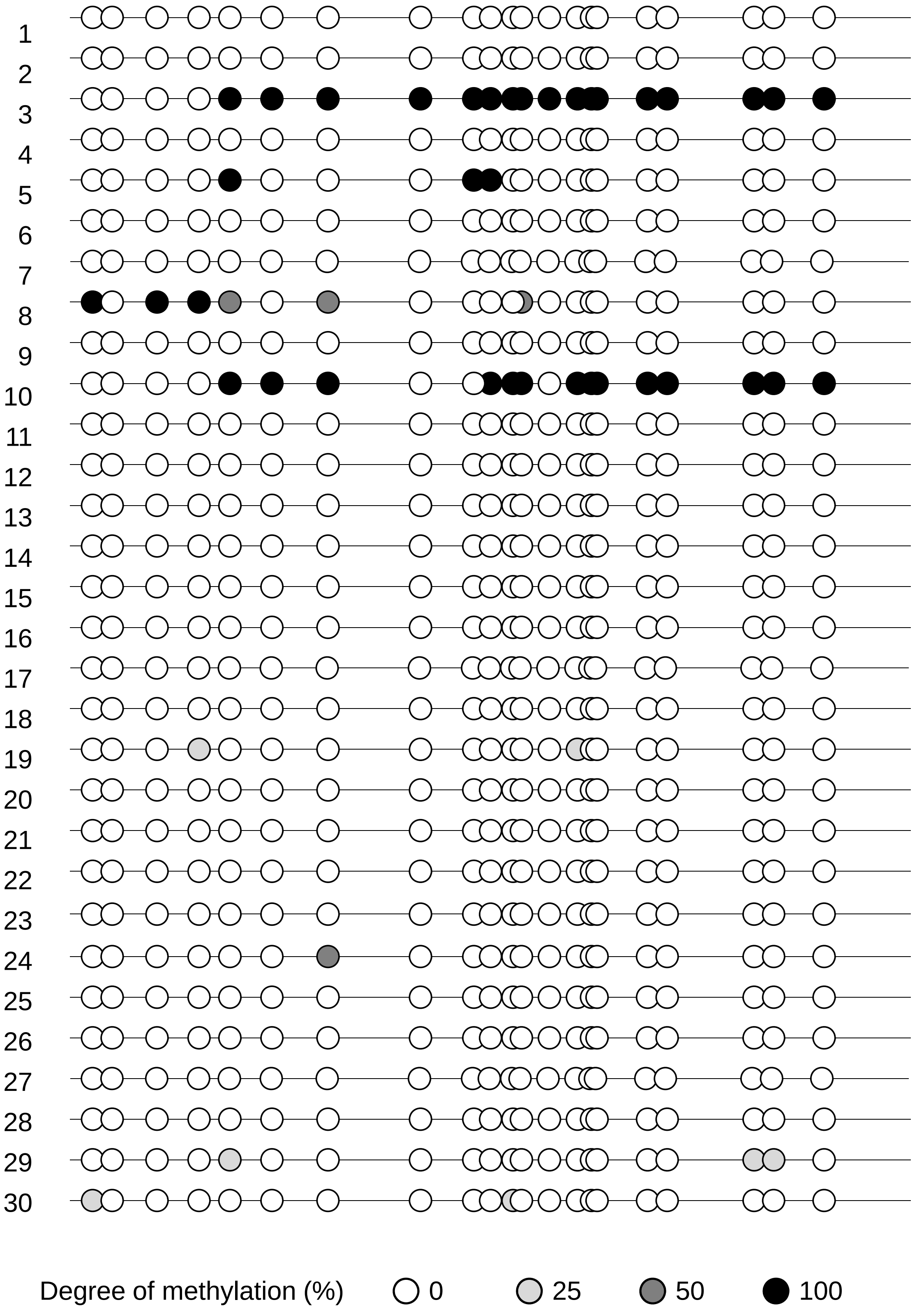

Supplemental Figure 8. Direct bisulfite sequencing data for EN1 CpG island promoter gene in the stool of 30 colorectal cancer patients.

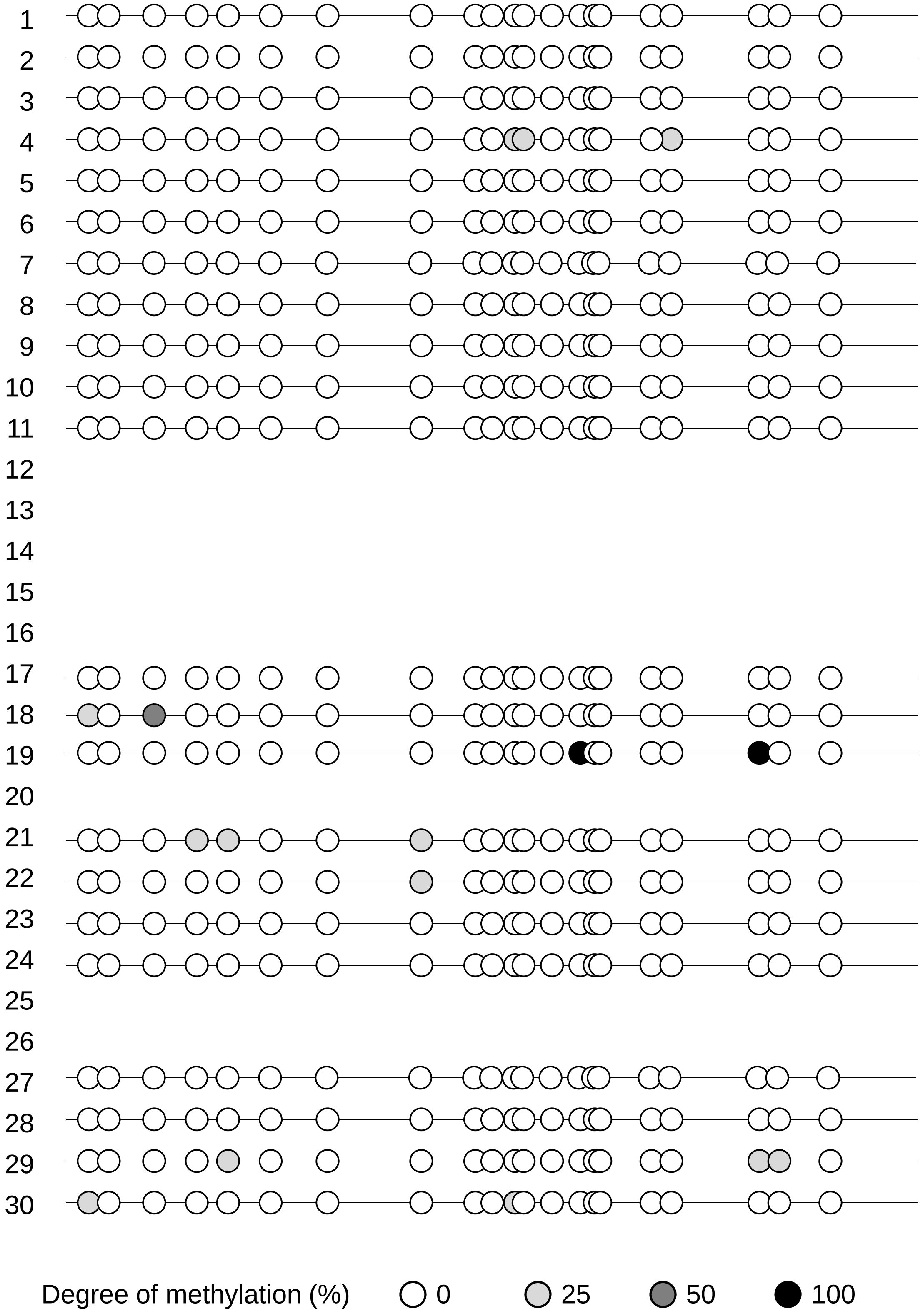

Supplemental Figure 9. Direct bisulfite sequencing data for EN1 CpG island promoter gene in the serum of 30 colorectal cancer patients.

Supplemental Table 1. Distribution of DNA methylation according to clinicopathological features of colorectal cancers

		n	EN1 M/U (%M)	n	SCTR M/U (%M)	n	INHBB M/U (%M)	No of methylated genes 0/ 1/ 2/ 3 (%)
**Categories**		90	66/24 (73)	90	48/42 (53)	91	23/68 (25)	9/36/37/9 (10/40/41/10)
**Sex**	**Male**	50	42/13 (84)	56	31/25 (55)	56	10/ 46 (18)	6/22/23/5 (12/44/46/10)
	**Female**	40	24/11 (60)	34	17/17 (50)	35	13/22 (37)	3/14/14/4 (7/35/35/10)
			p= 0.468		p= 0.667		**p= 0.049**	p= 0.970
**Dukes' stage**	**B**	42	36 / 7 (85)	43	28 / 15 (65)	43	14 / 29 (32)	1/14/20/8 (2/33/47/19)
	**C**	31	20/11 (64)	30	16/14 (53)	31	3/28 (9)	4/16/10/1 (13/51/32/3)
	**D**	16	10/6 (62)	17	4/13 (23)	17	6/11 (35)	4/6/7/0 (25/37/43/0)
			p= 0.102		**p= 0.015**		**p= 0.047**	**p= 0.020**
**Age (mean±SD)**		90	65±12/68±11	90	66±11/67±14	91	66±13 /66±12	69±12/67±14 / 65±11/66±14
			p= 0.463		p= 0.684		p= 0.984	p= 0.833
**Localization**	**Right**	26	22 / 4 (84)	26	14 / 12 (54)	26	11 / 15 (42)	1/8/12/5 (3/30/46/19)
	**Left**	64	44 / 20 (68)	64	34 / 30 (53)	65	12 / 53 (18)	8/28/25/4 (12/43/39/6)
			p= 0.188		p= 1.00		**P= 0.031**	p= 0.136
**p53 Mutation**	**Yes**	49	35 / 14 (71)	50	30 / 20 (60)	50	17 / 33 (34)	4/18/20/8 (8/36/40/16)
	**No**	29	22 / 7 (75)	28	13 / 15 (46)	29	5 / 24 (17)	2/15/11/1 (7/51/38/3)
			p= 0.794		p= 0.343		p= 0.126	p= 0.299
**K-ras Mutation**	**Yes**	31	26 / 5 (83)	30	16 / 14 (53)	31	9 / 22 (29)	1/11/17/2 (3/35/55/6)
	**No**	59	40 / 19 (68)	60	32 / 28 (53)	60	14 / 46 (23)	8/25/20/7 (13/42/34/12)
			P= 0.134		p= 1.00		p= 0.614	p= 0.158
**Microsatellite Instability**	**Yes**	7	5 / 2 (71)	8	3 / 5 (37)	8	3 / 5 (37)	1/4/2/1(12/50/25/12)
**(MSI)**	**No**	83	61 / 22 (73)	82	45 / 37 (54)	83	20 / 63 (24)	8/32/35/8 (10/38/42/10)
			p= 1.00		p= 0.465		p= 0.412	p= 0.827

Supplemental Table 2. Primer sequences for DNA methylation analysis.

Purpose	Primers sequence (5'- 3')
EN1 CpG island, bisulfite sequencing, melting curve analysis and MSP, first PCR	ACTATCCTACTTATAAACTC<br>AGAATAATAAAGATAAGAGAT
EN1 CpG island, bisulfite sequencing and melting curve analysis, nested PCR	CTACTTATAAACTCAACCAA<br>GTTTTAGGGATTTAGAGTTT
SCTR CpG island, bisulfite sequencing and melting curve analysis, first PCR	TCCAAAAACAATACTCTTTC<br>GTAGTAGTAGTTGTTGTAG
SCTR CpG island, bisulfite sequencing and melting curve analysis, nested PCR	CTAACCTCCTCCAACTTAAA<br>GTAGTAGTAGTTGTTGTAG
INHBB CpG island, bisulfite sequencing and melting curve analysis, first PCR	AAACCCTACCTCTATCCCAA<br>AAGTTTTGAGGTTTAGTGGTTTT
INHBB CpG island, bisulfite sequencing and melting curve analysis, nested PCR	CCTCCCCAACCAACAAAAA<br>GGTTAGGTTTTAGTGGTTATT
Methylated EN1 CpG island, MSP nested PCR	GTGGTCGTAGAGGTTAGGATC<br>CGCCATACCTTCTAACCCCGCG
Unmethylated EN1 CpG island, MSP nested PCR	GTGGTTGTAGAGGTTAGGATT<br>CACCATACCTTCTAACCCCACA
PTPN4 CpG island, bisulfite sequencing, first PCR	TTTGTAGGGAGGTAGGAGA<br>ACTTACCCCACCTAACTCA
PTPN4 CpG island, bisulfite sequencing, nested PCR	TTAGTAGTATGAGGTGGTGT<br>CCCAAAAACCACACATACAA
RALB CpG island, bisulfite sequencing, first PCR	AGGATTTTTTGGGGGAGTTT<br>CTTCCCCTTAAAAACCCCTT
RALB CpG island, bisulfite sequencing, nested PCR	TTTTTTGGGTTGTTTGGGGT<br>ACCAAAACAAAACCAACAAAC
TSN CpG island, bisulfite sequencing, first PCR	TTTTTTTTGTTTTTTTATGT<br>AAACAAATAATACTAAAAAA
TSN CpG island, bisulfite sequencing, nested PCR	TTTATGTTTAGTTGGGTTAT<br>AAATCAAAACAAAACAACAAA

Supplemental Table 3. Primer sequences for Real Time PCR expression analysis.

Genes	Forward primers (5'- 3')	Reverse primers (5'- 3')
EN1	TGGGTGTACTGCACACGTTATTC	CTTGTCCTCCTTCTCGTTCTTCTT
SCTR	CTCTGAAAGAAAGTACCTCCAGGG	GCAACAAAAATGGCTGGAGAA
INHBB	CGCGTTTCCGAAATCATCA	GGACCACAAACAGGTTCTGGTT
PTPN4	GATCTCCACCGGGAACTCCT	AACCGGGTTCCTTCCTGC
RALB	GAACATGAATCCTTTACAGCAACTG	CGACGAGCAGTGGAATTTTATCT
GLI2	CACCAGAATCGCACCCACT	GCCTGGGATCTTGCAGATGT
MARCO	GCTGCAGCGGGTAGACAACT	GCCTTGTTCACCTTTGATTCTGA
TSN	AGACCAAATTTCCTGCTGAACAG	AAGCGCTGCAACACAAACCT

Supplemental Table 4. Methylation of CpG island promoters in human colorectal cancer.

	**EN1 M/U [a]**	**SCTR M/U [a]**	**INHBB M/U [a]**	**No of Methylated genes 0 / 1 / 2 / 3**
**Normal tissue**	1/89 **(1)**	2 / 88 **(2)**	0 / 91 **(0)**	87 / 3 / 0 / 0 **(96 / 3 / 0 / 0)**
**Adenomas**	4 / 6 **(40)**	3 / 6 **(33)**	0 / 10 **(0)**	3 / 7 / 0 / 0 **(30 / 70 / 0 / 0)**
**Carcinomas**	66 /24 **(73)**	48/42 **(53)**	23 / 68 **(25)**	9 / 36 / 37 / 9 **(10 / 40 / 40 / 10)**
**X^2 / Fisher [b]**	p= 0.062	p=0.309	p=0.011	**p= 0.018**

[a] M/U: Methylated/Unmethylated (number in parentheses indicate percentage of cases exhibiting methylation)
[b] Comparison of Adenomas vs. Carcinomas using Fisher's or Pearson's Chi-square Test as appropriate.

Supplemental Table 5. Analysis of DNA methylation in EN1 in different samples from colorectal cancer patients using different techniques.

**Gene**	**EN1**									**Tumor clinicopathological features**			
**Technique**	**BS**			**MC**			**MSP**						
**Sample**	**T**	**ST**	**SE**	**T**	**ST**	**SE**	**T**	**ST**	**SE**	**Sex**	**Dukes' stage**	**Age**	**Localization**
ID 1	+	-	-	+	+	+	+	-	-	M	C	71	Sigmoid
2	+	-	-	+	-	-	+	-	+	F	D	67	Sigmoid
3	+	+	-	+	+	-	+	+	-	F	A	74	Rectum
4	+	-	-	+	+	+	+	-	-	M	B	71	Rectum
5	+	-	-	+	+	-	+	-	-	M	B	65	Rectum
6	+	-	-	+	-	-	+	-	-	M	C	87	Sigmoid
7	+	-	-	+	-	-	+	-	-	M	B	73	Sigmoid
8	+	+	-	+	+	-	+	-	-	M	C	55	Cecum
9	+	-	-	+	+	-	+	-	+	F	C	73	Cecum
10	+	-	-	+	-	-	+	-	-	M	ADE	83	Rectum
11	+	-	-	+	-	-	+	-	+	M	C	73	Left
12	+	+	-	+	+	-	+	+	-	M	A	77	Rectum
13	+	-	NA	+	-	NA	+	-	NA	M	A	80	Sigmoid
14	+	-	NA	+	-	NA	+	-	NA	F	ADE	71	Sigmoid
15	+	-	NA	+	-	NA	+	-	NA	M	D	64	Rectum
16	+	-	-	-	-	-	+	-	-	M	A	73	Cecum
17	+	-	NA	-	-	NA	+	-	NA	M	A	55	Left
18	-	-	-	+	-	-	+	+	-	M	B	65	Rectum
19	-	-	-	+	-	-	+	-	-	M	A	67	Rectum
20	-	-	-	-	-	-	+	-	-	F	C	88	Rectum
21	-	-	-	-	-	-	+	+	-	F	D	71	Rectum
22	-	+	-	+	+	-	+	-	-	M	C	73	Rectum
23	-	-	-	-	-	-	+	-	-	F	B	49	Splenic
24	-	-	NA	-	-	NA	+	-	NA	F	A	84	Left
25	-	-	NA	-	-	NA	+	-	NA	F	C	73	Rectum
26	-	-	NA	-	-	NA	+	-	NA	M	D	88	Sigmoid
27	-	-	NA	-	-	NA	+	-	NA	F	B	84	Rectum
28	-	-	-	-	-	+	-	-	+	F	D	76	Cecum
29	-	-	-	-	-	-	-	-	-	F	ADE	69	Sigmoid
30	-	-	-	-	-	-	-	-	-	F	C	65	Left
**Methyl.**	17	4(3)	0	18	8(8)	3(2)	27	4 (4)	4(3)				
**Unmethyl.**	13	26	21	13	23	19	3	26	18				
**Methyl %**	56.7	13.3	0	60.0	26.7	13.6	90.0	13.3	18.2				
**True+ %**		17.7	0		44.4	16.7		14.8	14.8				

T: tumor, ST: stool DNA, SE: serum DNA, BS: bisulfite sequencing, MC: melting curve analysis, MSP: methylation specific PCR. +: methylated, -: unmethylated. M: male, F: female. True positive (+) are methylated stool or serum DNA samples matching a methylated tumor sample as determined using the same technique.

Supplemental Table 6. Analysis of DNA methylation in EN1 in different samples from healthy controls using different techniques.

Gene	EN1							Sample information	
**Technique**	**BS**	**BS**	**MC**	**MC**	**MSP**	**MSP**			
**Sample**	**ST**	**SE**	**ST**	**SE**	**ST**	**SE**		**Sex**	**Age**
**ID** 1	+	-	-	-	-	+		M	53
2	-	-	-	-	NA	+		F	64
3	-	-	-	-	-	+		M	55
4	-	-	-	-	-	+		F	56
5	-	-	-	-	-	+		F	57
6	-	+	-	-	-	+		M	68
7	-	-	-	-	-	-		M	63
8	-	-	-	-	-	-		F	50
9	-	-	-	-	-	-		F	51
10	-	-	-	-	-	-		F	58
11	-	-	-	-	-	-		F	58
12	-	-	-	-	-	-		M	52
13	-	NA	-	NA	-	NA		M	69
14	-	NA	-	NA	-	NA		M	56
15	-	NA	-	NA	+	NA		F	66
16	-	NA	-	NA	+	NA		M	60
17	-	NA	-	NA	-	NA		F	49
18	-	NA	-	NA	-	NA		F	35
19	-	NA	-	NA	-	NA		M	60
20	-	NA	-	NA	-	NA		F	75
21	-	NA	-	NA	-	NA		F	62
22	-	NA	-	NA	-	NA		M	65
23	-	NA	-	NA	-	NA		F	74
24	-	NA	-	NA	-	NA		F	67
25	-	NA	-	NA	-	NA		M	59
26	-	NA	-	NA	-	NA		F	81
27	-	NA	-	NA	-	NA		M	68
28	-	NA	-	NA	-	NA		M	69
29	(-)*	NA	+	NA	NA	NA		F	65
30	-	NA	-	NA	-	NA		M	36
**Methylated**	1	1	1	0	2	6			
**Unmethylated**	29	11	29	12	27	6			
**False positive (%)**	3.3	8.3	3.3	0	6.6	50.0			

ST: stool DNA, SE: serum DNA, BS: bisulfite sequencing, MC: melting curve analysis, MSP: methylation specific PCR. +: methylated, -: unmethylated. M: male, F: female.
* Bisulfite sequencing of a fragment including 21 CpG sites revealed full methylation in 3 CpGs with full unmethylation in the rest, therefore it was considered as unmethylated.
